# The Staircase Drive—A Novel Actuator Design Optimised for Daisy-Chaining and Minimum Stress Load Coupling

**DOI:** 10.3390/s21227740

**Published:** 2021-11-20

**Authors:** Falk-Martin Hoffmann, Keith R. Holland, Nick R. Harris, Neil M. White, Filippo Maria Fazi

**Affiliations:** 1 Institute of Sound and Vibration Research, University of Southampton, Southampton SO17 1BJ, UK; krh.ex.isvr@gmail.com (K.R.H.); f.fazi@soton.ac.uk (F.M.F.); 2 School of Electronics and Computer Science, University of Southampton, Southampton SO17 1BJ, UK; nrh@ecs.soton.ac.uk (N.R.H.); nmw@ecs.soton.ac.uk (N.M.W.)

**Keywords:** actuator, piezoelectric, MEMS

## Abstract

This work presents a novel type of actuator that improves over the standard cantilever by permitting daisy-chaining while minimising stress to the joint connecting to the load. A detailed structural and functional comparison of the proposed device against the cantilever actuator as a baseline is given, led by a brief revision of the cantilever actuator as the state-of-the-art that highlights its limitations with respect to daisy-chaining and the stress it inherently creates within the joint connecting to the load when attempting out-of-plane displacement without rotation. Simulations of both devices’ performance confirm that the newly proposed device yields the targeted displacement profile that both enables the daisy-chaining of such a device into a higher-order actuator for increased displacement and reduce stress in the joint with the load. This comes at the cost of reduced maximum displacement compared to the cantilever, which can be overcome by daisy-chaining. The proposed device’s performance is further evaluated on the basis of manufactured prototypes measured by means of a laser scanning vibrometer. The prototype was manufactured on a 150 μm alumina substrate, and both electrodes and piezoelectric layer were deposited in a thick-film printing process.

## 1. Introduction

Microelectromechanical System (MEMS) actuators are enjoying a lot of industrial interest, with applications ranging from micro-mirrors [1,2] (e.g., for use in video projectors) over energy harvesting devices [3,4] and micro-speakers [5,6,7,8]. The deformations/translations of MEMS devices occurring upon when actuated can vary in nature, depending on the application, e.g., some devices displace, and some twist.

Currently, many of the aforementioned applications rely on cantilever actuators as the basic element of the actuation. This actuator relies on the deformation of a cantilever by bending forces that uniformly shear the cantilever in one direction. A detailed description of an early piezoelectric cantilever microactuator was given by DeVoe and Pisano [9]. This basic design has recently been used in a range of configurations that deviate from its purest form, e.g., curved [5], concatenated [2,10], and with different transduction mechanisms (e.g., piezoelectric [5] and electrostatic [10]). However, ultimately, either design is yielding a deflection of the tip of the cantilever from the plane within which the fixed end and the corresponding profile’s tangent lies. The tip itself ends up in a different plane that is *not parallel* to the fixed end’s plane. This can be useful and is exploited for some applications (see, e.g., Reference [3]). However, it will be shown in this work that this also has other implications that may be undesirable for certain applications, e.g., when the load does not permit rotation while displacing or in order to daisy-chain two actuators.

This work proposes a novel ‘staircase’ actuator that yields a deflection of the tip of the actuator such that the tangent plane of the tip is parallel to the plane of the fixed end. Its advantages are derived from this inherent nature of the device, overcoming the aforementioned problems of the cantilever actuator and permitting the creation of higher order actuators by daisy-chaining more than one unit. This study reports the theoretical concept behind the invention, simulation work, confirming the targeted performance and comparing the proposed device to the state-of-the-art, a detailed route to manufacturing, and experimental confirmation of the technology based on measured data obtained from prototypes.

The remainder of this work is organised as follows. First, the state-of-the-art cantilever actuator based on piezoelectric actuation is revisited, with a focus on inherent shortcomings that led to the discovery of the novelty presented in this work. The following section presents the novel staircase actuator that features a special displacement profile with a net zero difference between the tangent angles at the beginning and the end of the actuator. This permits the concatenation of two or more actuators into a newly defined higher order actuator device. It is shown by simulation that the implementation of such a higher order actuator is impossible with the state-of-the-art cantilever actuator. The Section 4 then presents the manufacturing process used to create the prototype staircase actuator. The Section 5 confirms the theoretical considerations and simulations by means of laser vibrometer measurements of the created actuator. Here, the targeted displacement profile is shown by graphical reconstruction of the displaced device from measured data, and the dynamical behaviour of the actuator is described for three implementation of the prototypes. In the final section, the findings are concluded.

## 2. The Cantilever Actuator

### 2.1. The Structure

**Figure 1:**
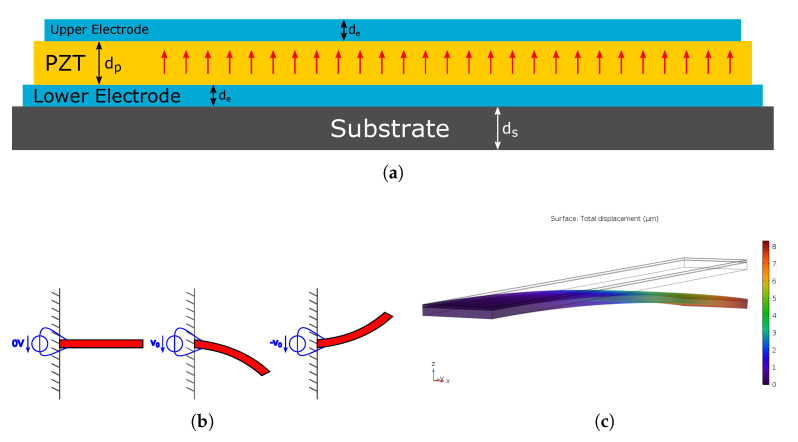
(**a**) Layered structure of the piezoelectric cantilever actuator with indicated polarisation direction (red arrows), (**b**) resting position and cantilever flexural modes, and (**c**) COMSOL simulation of the displaced cantilever (displacement exaggerated).

Figure 1 shows the structure of a cantilever actuator with piezoelectric actuation principle.

The homogeneous, non-isotropic piezoelectric material, e.g., Lead Zirconate Titanate (PZT), is sandwiched between two electrodes, and the combined structure is supported by an underlying substrate. The PZT is polarised in the direction normal to the plane of both electrodes.

### 2.2. The Deflection Profile of the Cantilever

**Figure 2:**
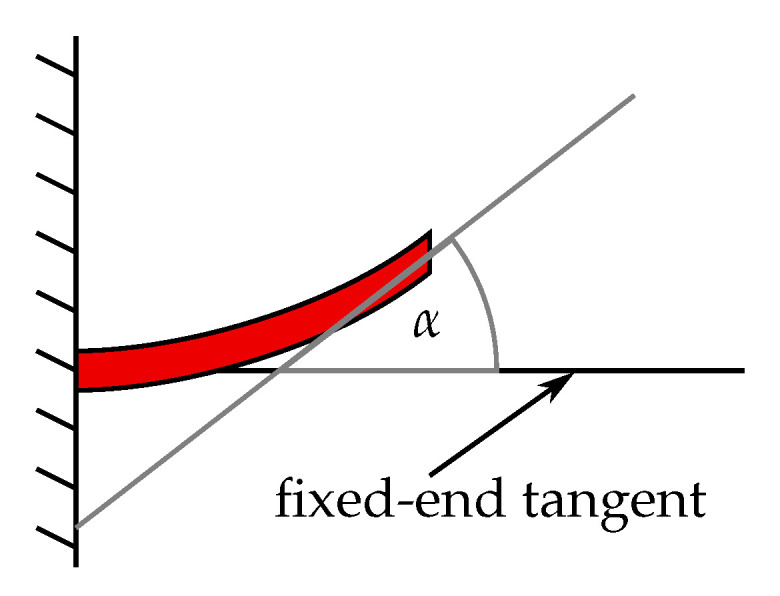
Drawing of the tangent angle α to the horizontal plane at the furthest point from the cantilever’s suspension.

Applying a voltage to the two electrodes evokes a piezoelectric effect, the strength of which is determined by the $d_{31}$ coefficient of the material. The contraction or expansion of the piezoelectric material, depending on the sign of the applied voltage, yields a flexural mode deflection of the cantilever, as shown in Figure 1b. Since the contraction (or expansion) of the piezoelectric layer would theoretically occur homogeneously across the full area, the resulting flexural mode has constant curvature. As a consequence, the largest deflection of the beam is determined by the $d_{31}$ coefficient and the length of the cantilever, and it occurs at the point farthest from its suspension. Therefore, assuming a horizontal resting position of the cantilever, the tangent to the beam at the farthest point from the suspension has the largest *tangent angle* to the horizontal plane, α, as shown in the drawing in Figure 2.

**Figure 3:**
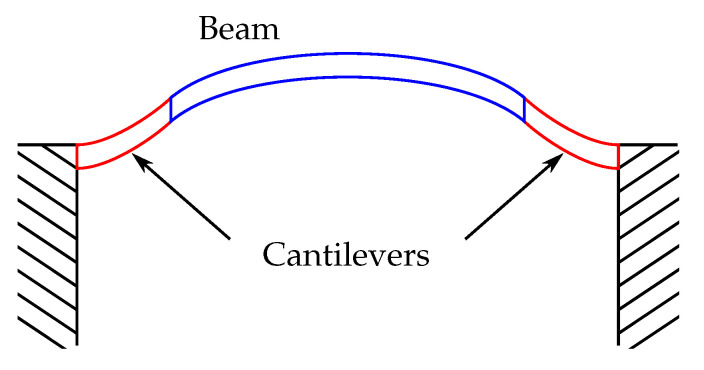
Beam (blue) bending due to the induced stress induced by the displacement of the connected two cantilever actuators (red).

However, if the cantilever is intended as a actuator for an object attached at its end point through a joint, this may pose a problem. At large displacements, the resulting large angle α can introduce a significant stress to the joint or even the object attached to the cantilever, if the bond is comparably rigid. A scenario for such a case is depicted in Figure 3, where a long beam is connected to two cantilever actuators at its ends.

It can be understood that the flexural mode in the actuators and the resulting tangent angles induce a (often undesirable) bending of the beam through the rigid joint (not depicted) in between. Additionally, stress in the joints and the beam is introduced.

**Figure 4:**
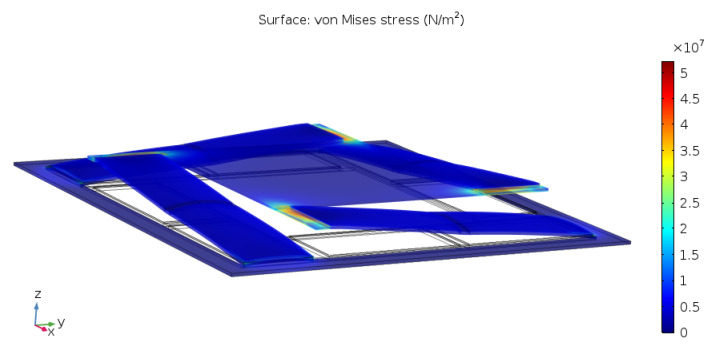
von Mises Stress in the joint connecting the end point of the cantilever actuator to the load.

To illustrate this hypothesis, a scenario with four cantilever actuators moving a square plate was simulated in COMSOL. The result for the stress in the joints between the end point of the beams and the connected load is shown in Figure 4.

Red colour indicates regions of high stress. It is evident that the joints have become a potential weak point of the structure.

Depending on the substrate material, the induced stress may not actually be a problem. In the course of this work, both alumina and stainless steel have been used as substrate material, where the former is known to be brittle in nature. Anyway, neither of the two were causing problems related to this stress with the designs investigated. However, this and the impact of displacement on the strength of the stress remain subjects of future work. Nevertheless, the mere existence of this non-zero ‘contact angle’ causes another significant limitation of the cantilever actuator that is now investigated in more detail.

Let a target device based on the cantilever actuator have fixed target dimensions and displacement requirements. If the peak displacement cannot be achieved by further increasing $d_{31}$, a sufficiently long cantilever is required instead. However, in the case that the required cantilever length exceeds the target dimensions of the device, the only solution may be a different actuator shape to increase the length of the cantilever while maintaining the same footprint, e.g., the arc-shaped actuator proposed by Arevalo and Foulds [5].

However, even a curved shape of the cantilever may not gain enough length to meet the displacement requirements. Or even when it does, it may introduce undesirable side effects. It would, therefore, be helpful if one could daisy-chain, i.e., concatenate several shorter cantilever actuators and arrange them in a meander to meet the target while solving the length-related issue. However, it is shown in the following subsection that this is not achievable with cantilever actuators due to the *non-zero tangent angle*.

### 2.3. Daisy-Chaining Cantilever Actuators

The daisy-chaining of actuators aims to combine their individual displacements to a proportionally larger displacement of the joint structure. To refer to such devices more specifically, the following definition shall be used in the remainder of this work:

**Definition** **1.** *A higher-order actuator is the concatenation of two or more identical actuators to form a single device: i.e., a 2nd-order actuator is composed of two combined 1st-order actuators, and a 3rd-order actuator is composed of three 1st-order actuators and so forth*.

**Figure 5:**
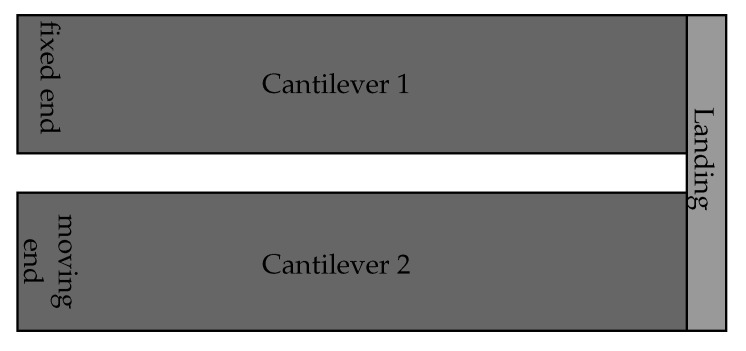
Top view of a 2nd-order cantilever actuator, with the anchored actuator at the top, the landing on the right, and the second actuator at the bottom.

The lack of suitability of the cantilever actuator for daisy-chaining can be demonstrated by considering the simplest case of a 2nd-order cantilever. With the objective to reduce the length of the combined device, it makes sense to arrange the two cantilevers in parallel and connect them in the same way that two flights of stairs are connected by a landing. The top view of such an arrangement is shown in Figure 5, where the joint connecting the cantilevers shall be referred to as the landing.

With this arrangement, the goal is to create a larger displacement with respect to the plane within which the fixed-end tangent lies. Simulating the displacement of the arrangement, however, reveals that the intended behaviour is in fact not achieved.

**Figure 6:**
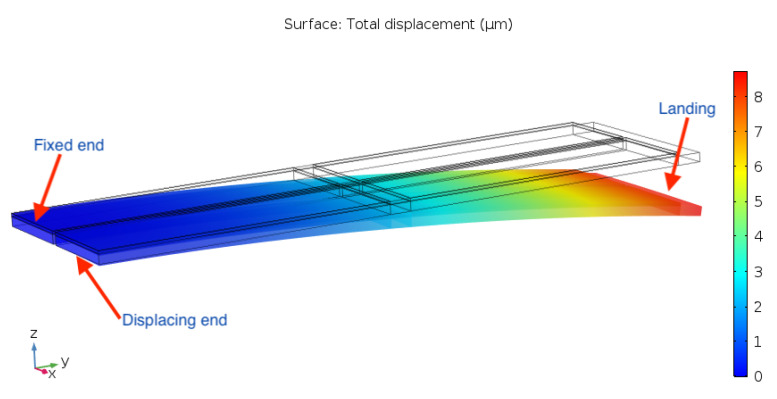
Second-order cantilever actuator flexural displacement.

From Figure 6, it can instead be seen that the displacement profile’s curvature turns back on itself, i.e., the moving end comes back to the same position along the *z*-axis as that of the fixed end. As a consequence, the largest displacement in the *z*-direction is obtained at the end of the landing, giving the device a comparable performance to the 1st-order cantilever.

**Figure 7:**
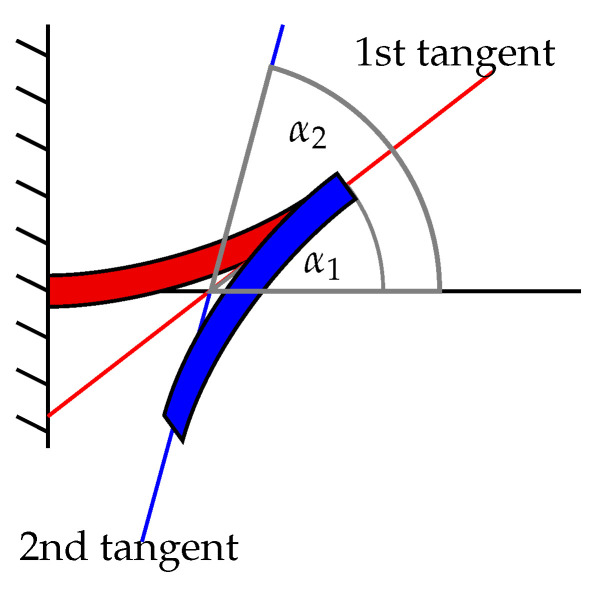
Definition of the first and second tangent angle, $\alpha_{1}$ and $\alpha_{2}$, respectively, with respect to the 2nd-order cantilever actuator.

One could argue that, by driving the second cantilever in opposite phase to the first, the achievable point-to-point distance between the fixed end and the displacing end would be much larger than the vertical displacement of a single cantilever. While that may be true, it also results in a significantly larger tangent angle $\alpha_{2}$ at the displacing end (see Figure 7).

In fact, it is straightforward to show that $\alpha_{2}=2\alpha_{1}$, assuming that both cantilevers are identical and that the landing does not twist due to the forces induced by the actuators. Consequentially, even though the potential displacement obtained by this variant is improved, the original requirement for a zero total tangent angle is not met.

As a consequence, the 2nd-order cantilever is unable to meet simultaneously the requirement to increase the device’s net displacement compared to the 1st-order cantilever and also yield a zero tangent angle at the displacing end. Equally, higher order actuators of order larger than 2 will ultimately also suffer from the same problem, indicating that the problem is inherent to the cantilever actuator.

To overcome this limitation and make the potential of the higher order actuator available, a novel actuator principle is introduced in the next subsection that addresses this issue.

## 3. The Staircase Actuator

**Figure 8:**
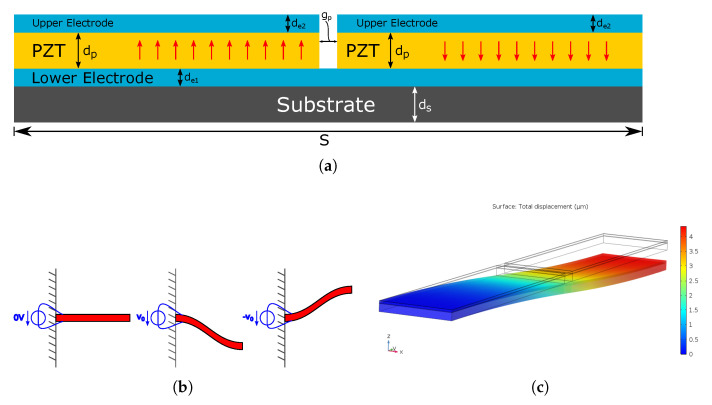
(**a**) Layered structure of the piezoelectric cantilever actuator with indicated polarisation direction (red arrows), (**b**) resting position and staircase flexural mode showing when applying different voltages between upper and lower electrode, and (**c**) COMSOL simulation of the displaced cantilever (displacement exaggerated).

The non-zero tangent angle at the displacing end of the cantilever is a natural consequence of its shape when actuated. While the gradient of the curvature on the cantilever is zero, i.e., the curve progresses constantly, it is necessary to introduce an abrupt change of the sign of the curvature in order to bend the beam back towards a zero tangent angle at the displacing end. Any kind of abrupt change is unwelcome; so, an alternative is to provide a more controlled change of direction of slope by effectively making two beams in series. Starting from a piezoelectric cantilever actuator, this can be achieved by interrupting the piezoelectric layer halfway along the beam and polarising the two separate piezoelectric patches with opposite signs, as shown in Figure 8a.

**Figure 9:**
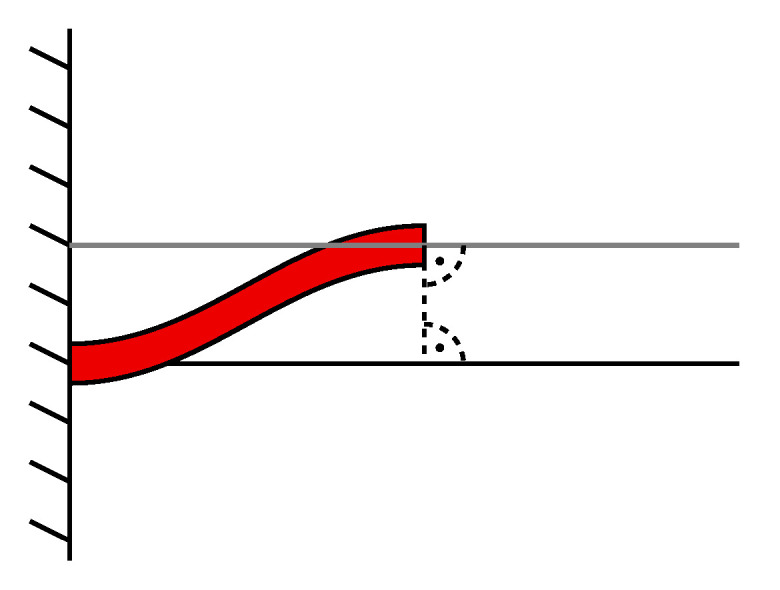
Drawing of the tangent angle to the horizontal plane at the farthest point from the staircase’s suspension.

When then applying the same positive voltage to both, the piezoelectric patch closer to the suspension will laterally expand, thus bending the beam downwards, while the other patch will laterally contract and create a curvature in the second half of the beam that opposes that of the first half. If both piezoelectric patches have the same size and strength of polarisation, the resulting tangent at the farthest point from the suspension is then parallel to that when they are in the resting position, as depicted in Figure 9.

### 3.1. Advantage of the Staircase Actuator

The implications of the fact that the tangent at the end of the staircase is parallel to the resting position shall be considered closer in the following. Naturally, the net displacement at the endpoint of the staircase is less than that of the original single cantilever, since half its length is required to counter the curvature introduced by the first half. This can be seen when comparing the maximum displacement in Figure 1c and Figure 8c, where it shows that the cantilever actuator reaches about twice the displacement of the staircase actuator.

**Figure 10:**
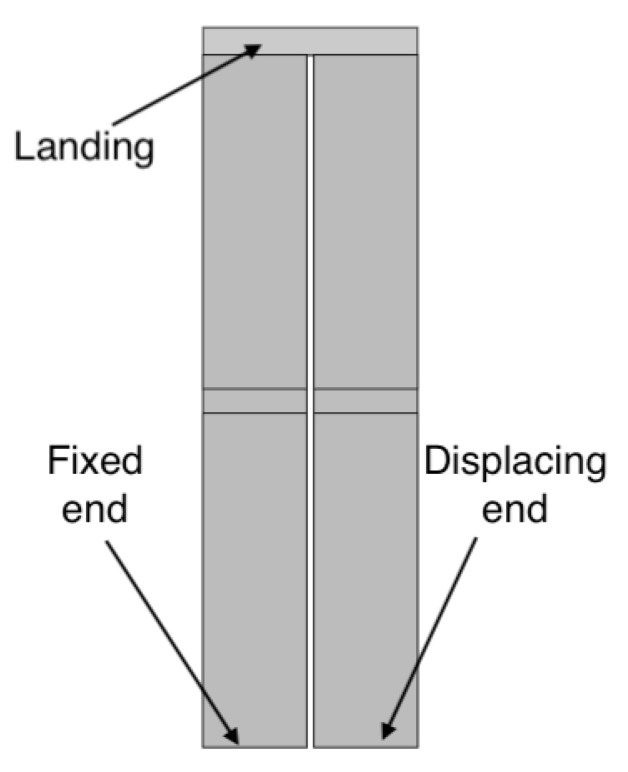
Top view of a 2nd-order staircase actuator, with the anchored actuator on the left, the connecting landing at the top, and the second actuator on the right.

However, unlike the cantilever actuator, the staircase actuator reaches a horizontal tangent at its endpoint and can, therefore, be joined with another staircase actuator. The product would be a 2nd-order actuator that consists of two staircase actuators (i.e., two 1st-order staircase actuators) chained together. The numeral of the order, therefore, represents the number of connected staircase actuators. The top view of a 2nd-order arrangement is shown in Figure 10.

**Figure 11:**
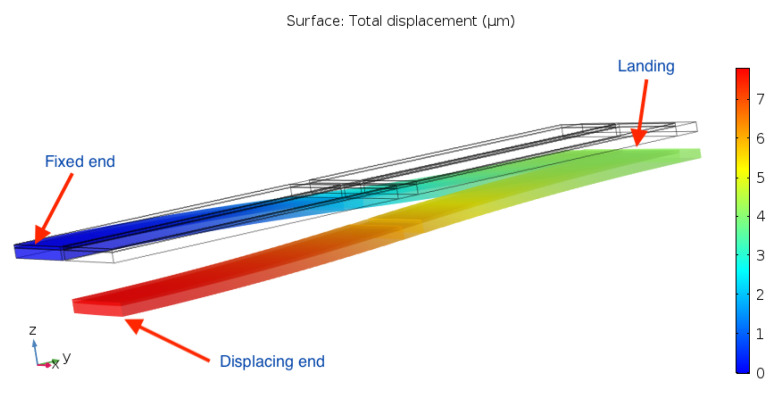
2nd-order staircase actuator’s flexural displacement, where comparison to Figure 6 shows that the cantilever yields zero net start-to-endpoint displacement and the staircase doubles its start-to-endpoint displacement compared to a 1st-order device.

Note that both staircases run parallel but with a lateral shift, exactly as in a multi-floor staircase in a building. The advantage of the 2nd-order staircase actuator is that it almost doubles the net displacement of the 1st-order staircase actuator (see Figure 11).

The important result for the 2nd-order staircase actuator entails that these novel actuators can be daisy-chained in parallel to increase the maximum displacement with every subsequent actuator. It can also be seen that a 2nd-order staircase actuator approximately matches the net displacement of the 1st-order cantilever actuator. However, the cantilever actuator cannot be extended usefully beyond order 1. The option to use staircase actuators and create higher order actuators is a major distinction from prior art and a novelty of the proposed actuator design.

**Figure 12:**
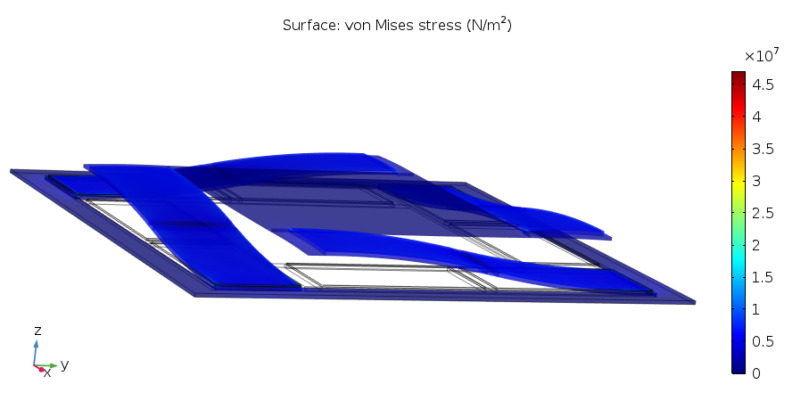
von Mises Stress in the joint connecting the end point of the staircase actuator to the load.

On top of the possibility of higher order devices, it was discovered that the staircase actuators also bear the potential to induce comparably less mechanical stress to the joint connecting the actuator to the load. The result of the COMSOL stress analysis for the joint connecting the staircase actuator to a load (compare to Section 2) is shown in Figure 12.

It can be seen that, compared to the cantilever actuator and for a load that does not permit rotation with respect to the xy-axis, the stress induced by the staircase actuator is considerably lower. This is also a novel and useful result adding further to the potential for this configuration.

Since the staircase actuator with its two sections per devices naturally has more design parameters than a cantilever, the most essential of these shall now be briefly described.

### 3.2. Staircase Actuator Design Parameters

The dynamic performance of the actuator is dependent on the physical properties of the materials used to construct it, as well as various dimensions that are adjustable. Figure 8a illustrates some dimensions, and all the key variables in the modelling that follows are listed here:

- order *n*,
- actuator length *S* (m),
- actuator width *L* (m),
- substrate thickness $d_{s}$ (m),
- electrode thicknesses $d_{e1}$ and $d_{e2}$ (m),
- piezoelectric layer thickness $d_{p}$, and
- the gap between the piezoelectric patches $g_{p}$.

These parameters determine the weight and flexibility of the actuator, which naturally also have an impact on the performance. Note that, in an ideal device, all layers cover the full length (minus $g_{p}$) and full width *L* of the staircase. To maximise the displacement, the size of the gap $g_{p}$ should be kept as small as possible. Furthermore, if the sandwich of electrodes and PZT layer do not extend across the full width *L* of the cantilever, the efficacy may also then be adversely affected.

Since these parameters pertain to the physical dimensions of the device, some of these factors may also be determined and/or limited by the underlying manufacturing process, which is outlined in the subsequent section.

## 4. The Manufacturing

The overall manufacturing process of the proposed MEMS transducer must be tailored to serve the special requirement of the staircase actuator to have its two patches of PZT polarised in opposing directions. In this work, this was achieved by polarising the PZT AFTER the assembly of the layered structure.

The full process is organised in five stages:

1. thick-film printing,
2. laser cutting,
3. bonding device to Printed Circuit Board (PCB),
4. wire bonding for polarisation,
5. polarisation, and
6. wire bonding for driving operation.

Each stage is described in the following subsections.

### 4.1. Thick-Film Printing

The thick-film structure of the device is created through a screen-printing process where specialised inks of the individual layers’ material are deposited sequentially onto a suitable substrate. This work makes use of a bespoke PZT cermet ink developed at the University of Southampton [11]. In order to achieve good performance, each layer is sequentially printed then fired at 850 ${}^{\circ }C$ before the next layer is added. A good thickness of the conductive layers (gold or maybe low-migration silver palladium) has been previously established to range from 2 μm to 5 μm, whereas good results have been achieved with PZT layers in the range 40 μm to 70 μm. There is scope for further investigation into this parameter, as lower values may be possible to reduce the voltage required for the same electric field strength.

Since the lower electrodes are all connected to ground, printing onto a conductive substrate is non-critical and does not require an insulator.

**Figure 13:**
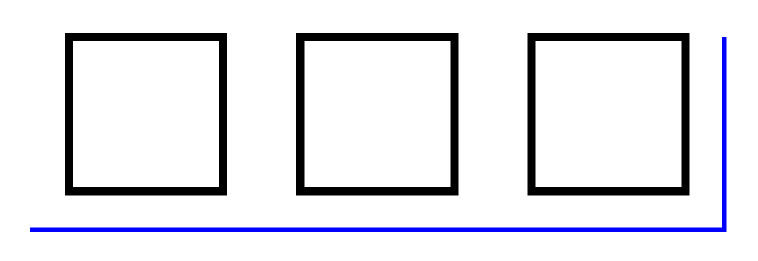
Laser cutting features (blue) to aid alignment of the laser cutter when cutting out the transducers (indicated as black squares).

A consequence of the transducer structure, further features are deposited during the printing process. With features as small as 200 μm, the manufacturing requires high precision which must be preserved between both the printing and the cutting process. In order to align the printed substrates on the laser cutting table, additional alignment features in the form of lines with the width of the laser’s cut width are printed onto the substrate (see Figure 13).

The alignment process is described in detail in the next section.

### 4.2. Laser Cutting

The second step of the manufacturing process is to cut the transducers from the encompassing substrate. This process is based on a CO${}_{2}$ laser and a Computer Numerical Control (CNC) table holding the substrates. Each device is cut out individually.

While a range of substrates can be used with the thick-film processing techniques, the process is well-suited for alumina ceramic substrates but certain steels are also compatible. With alumina, the laser cutting process is straightforward. However, with steel, it was found that another layer of steel below the actual substrate as a sacrificial layer draws most of the burr occurring on the laser’s far side. With either material, any residual burr is removed by gently rubbing the cut-out samples along the surface of a perforated sheet of alumina.

One aspect that that is important for this part of the manufacturing process is the issue of alignment. To facilitate a good alignment of the cuts with respect to the thick-film structure, all prints are aligned to one specific corner of the substrate. To further improve the accuracy, additional alignment features in the form of thin lines are printed onto the substrate (see Figure 13). These lines are sacrificial and serve an iterative alignment process where the laser is calibrated incrementally along both the *x* and *y*-axis until it cuts the line perfectly in the middle.

The device is cut from the substrate with an outside frame of about 1 mm in width (e.g., as shown in Figure 12). This frame is needed to anchor the staircase arms and to bond the full device to a suitable carrier (e.g., a PCB board with connection pads).

Once cut, any remaining burr must be removed from the edges of the arms to ensure the actuators can move freely. Now, the device is ready for bonding to the PCB.

The laser cutting also revealed a side-aspect of the manufacturing process and the chosen material. The devices built on the basis of a steel substrate were very likely to display light warping, while the devices based on alumina were not showing signs of warping. The huge temperature variations during the printing process and the differences in the heat expansion coefficients of the different materials involved is deemed to be the cause of this, along with the generally higher stiffness of alumina.

### 4.3. Assembly onto PCB

**Figure 14:**
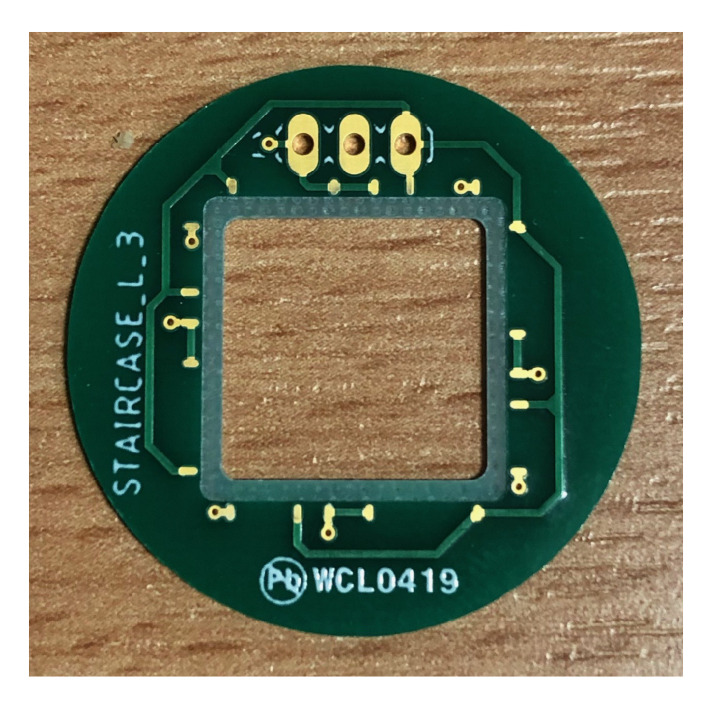
PCB specifically designed to hold the device with frame, staircase actuators, and diaphragm.

In order to make the mechanically delicate device more manageable for this work, it is glued onto a specifically designed PCB. It features a square gap the size of the active dimensions (i.e., the moving part) of the device, so that the diaphragm and staircase arms can move freely up and down (see Figure 14).

The cutout is surrounded by a milled area that is set back by 200 μm with respect to the top of the PCB, so that the cut-out device with its frame fits nicely onto this pedestal. To fix the device to the PCB, glue is applied to the top face of the milled area, onto which the device is then mounted. Given the brittle nature of the material, the devices created on alumina substrates had to be handled with greater care, while the steel substrates have been less critical about the mechanical handling.

Once the glue has set, the transducer is ready for the wire bonding process.

### 4.4. Wire Bonding for Polarisation

The wire bonding process serves to create interconnections between the staircase arms and the driver circuit on the PCB board. This brings unique challenges to the traditional wire bonding process, the two major ones being bonding to a flexible surface and bonding to a printed layer. These are briefly outlined in the following.

Wire bonding requires the bonding surface to be mechanically secure so that the applied ultrasonic forces can be entirely used for the bonding process. Due to the flexible staircase arms, they are likely to give in to the applied force or even get damaged. To avoid this, the device must be supported on its underside by temporarily filling the gap in the PCB with a bespoke support made from shim material that maintains stability in its shape at a bonding temperature of 65 ${}^{\circ }C$. This temperature is required as part of the wirebonding process. Ideally, the support prevents both lateral and vertical movement during the bonding process.

The printed metallic electrode consists of metal particles suspended in a glass binder, which poses a challenge since it is easier to form intermetallic bonds with a layer of bulk metal. Wire bonding is a welding process which relies on diffusion of metallic particles/electron sharing, so the binders in the printed electrode interfere with the metallic bonds. In combination with comparably low bonding temperature and a potentially flexible substrate, the risk of a bond to fail is high. This was overcome by increasing the ball size for the first bond and ensuring the use of optimum ultrasonic power. A higher than optimum power would lead to craters in the printed layer and a lower than optimum power would lead to attachment failures of the first bond. Furthermore, each interconnection can be reinforced by making a security ball over the wedge bond, and 25 μm gold wire was used for wire bonding the actuator assembly.

**Figure 15:**
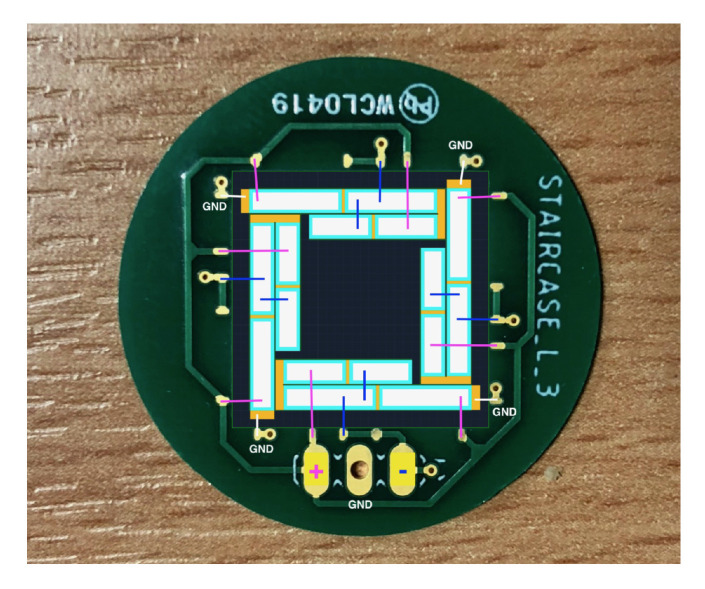
Wiring scheme for a 2nd-order transducer, with ground connection in white, positive polarisation voltage in magenta, and negative polarisation voltage in blue.

The interconnection scheme for polarisation is different than for driving, as the ‘upper’ PZT patches of the staircase actuator must be polarised in the opposite direction to that of the ‘lower’ patches. Therefore, they need to be addressed with polarisation voltages of opposite sign with respect to ground. The full wiring scheme for a 2nd-order device is shown in Figure 15.

The PCB features two interconnection pads at each corner of the device; one serves to provide the positive polarisation potential (and later the driving signal) and one to serve as ground. However, for the polarisation process, the PCB also features pads along the middle of each edge. These pads are connected to negative polarisation potential only.

With all wire bonds in place, the device can now be polarised.

### 4.5. Polarisation

The magnitude of the polarisation voltage must be matched to the thickness of the PZT layer for optimal performance and device yield. The overall field strength should be in the range of 4 MV$m^{-1}$ to 8 MV$m^{-1}$.

The polarisation voltage should be applied for as long as possible to maximise the effect. Furthermore, the device should be heated to approximately 120 ${}^{\circ }C$ while being polarised. Before turning the polarisation voltage off, the temperature of the device must be dropped to below 50 ${}^{\circ }C$ again.

Once the polarisation is completed, the wiring scheme can be changed to that for driving. Note that, in a more automated manufacturing process, the polarisation can be achieved with spring-loaded needle contact to the pads, so that wire bonding is only required for the driving operation, as described in the next subsection.

### 4.6. Wire Bonding for Driving Operation

**Figure 16:**
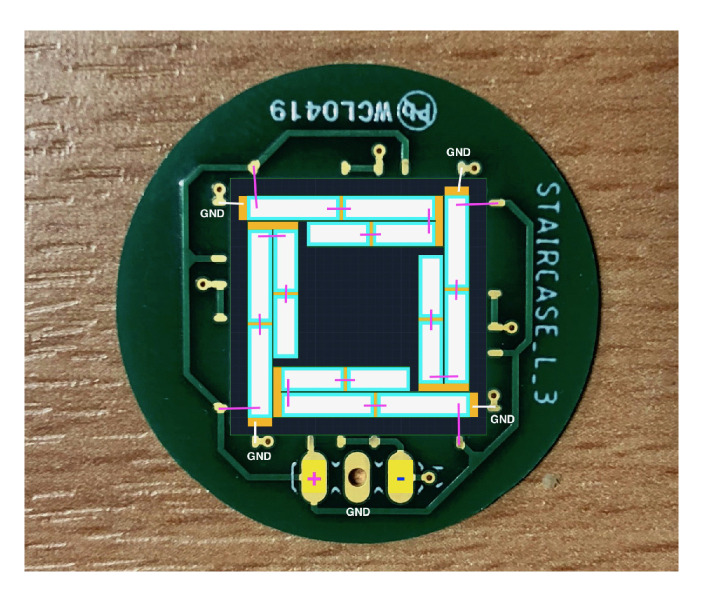
Wiring scheme for a 2nd-order transducer for driving operation, with ground connection in white, and signal connections in magenta.

The wiring scheme for driving operation must be realised, as shown in Figure 16.

All upper electrodes of the patches are daisy-chained for each actuator, enabling parallel actuation.

At this point, the device is fully operational.

## 5. Vibration Measurements

To verify the functioning of the proposed actuators and to confirm the creation of the targeted displacement profile that enables the creation of higher-order actuators, samples with the design parameters as outlined in Table 1 were measured: one sample from alumina substrate (Type 1), and two samples from steel substrate, with different beam width *L*, (Type 2 and 3).

**Figure 17:**
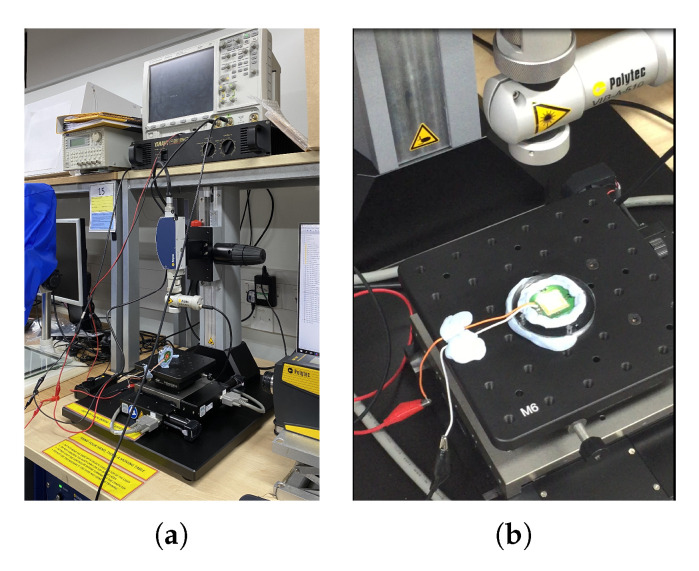
(**a**) Overview and (**b**) moving table with device under test of the vibration measurement setup.

All manufactured devices were analysed using a *Polytec MSA-050 Micro System Analyser* for high-precision scanning laser vibrometry to capture the out-of-plane motion of the devices. The MEMS devices were mounted on a perspex flange to increase the volume and compressibility of the air cushion between the actuators and the moving table, to avoid influencing the resonance frequency of the device. The assembly was then laid on the moving table of the vibrometer. The drive voltage with a peak amplitude of 40 V was delivered to the electrodes of the device under test (DUT) through a YAMAHA P2160 power amplifier. Figure 17 show the vibration measurement setup with the DUT.

**Figure 18:**
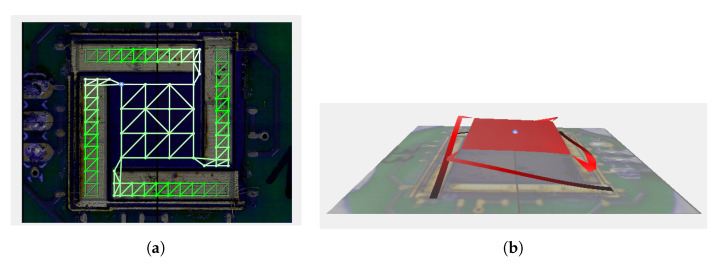
(**a**) Scanning points use for vibration measurements. (**b**) Displacement profile reconstruction of the Type 3 device at 400 Hz.

The measurement test signal was a periodic chirp, from 100 Hz to 20 kHz, generated by the *Polytec* measurement system. The latter captured the input voltage to the DUT (using a custom-made voltage divider to bring the voltage delivered by the *YAMAHA* power amplifier down to the corresponding dynamic range and avoid clipping) and the velocity normal to the surface of the device, measured at a number of points defined on a measuring grid. This grid was defined to capture all relevant vibrating parts of the device under test and is reported in Figure 18a.

From the combined measured signals, the system then computed the transfer function between input voltage and acceleration, i.e., the level of acceleration per voltage fed to the electrodes, for each of the points on the measurement grid. As a confirmation of the staircase shape that is to be obtained when driving the actuators, Figure 18b shows a reconstruction of the displacement profile from the measured data. The result shows the expected profile where the upper half of the actuator is bending the opposite way as the lower half. Note that, due to the limitations of the measurement system, this profile had to be measured dynamically, i.e., with the device in motion but below its resonance but not statically displaced.

**Figure 19:**
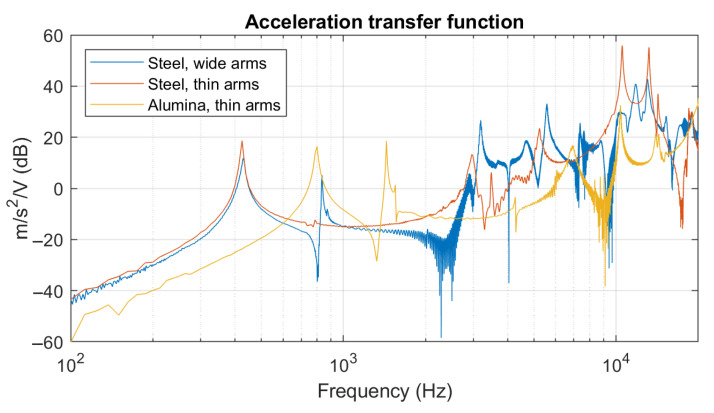
Acceleration transfer functions measured at the centre of different devices.

The acceleration transfer functions measured at the central point of three different type of devices are reported in Figure 19.

It can be observed that the two steel devices (i.e., Type 2 and 3) have the same main resonance just above 400 Hz, even if they have a different surface ratio between arms and piston, due to the difference in actuator (In the figure’s legend, this is denoted as ‘arm’.) width *L*. The two devices with thin arms, on the other hand, have a peak at about 10 kHz. This peak is also present but less pronounced in the device with wide arms. The alumina device (Type 1) shows the clear distinction of a higher resonance frequency, compared to the steel devices. This suggests that the material and its compression modulus have a substantial influence on the actuators stiffness, whereas only a minor influence is attributed to the actuator’s width.

## 6. Conclusions

Based on the piezoelectric cantilever actuator, a novel staircase actuator design was proposed. The novel device features two instead of one contiguous patches of PZT. The two PZT-sections are polarised with opposing signs to yield an S-shaped displacement profile. This innovation enables the creation of higher-order motors through concatenation and potentially reduces stress to the joint connecting the motor to the load, when a rotation of the load is not possible. It was explained that the derived higher-order devices can, in principle, yield an arbitrary maximum displacement from the same drive voltage as the single-order device.

The device and its design parameters were described in detail and distinguished from the state-of-the-art. Simulation results were given to compare the new actuator to the cantilever actuator, confirm the targeted displacement profile predicted from theoretical considerations, and present the advantages and the drawbacks of the design. It was argued that, while a single staircase actuator has a lower maximum displacement than a cantilever actuator, a 2nd-order staircase actuator is already on par and a 3rd-order design would outperform the cantilever actuator, which cannot be concatenated as it was shown. Furthermore, the predicted reduction of von Mises stress occurring in the joint that connects the novel actuator to a load that does not permit rotation was confirmed by simulation, comparing to the performance of the state-of-the-art.

The manufacturing process based on thick-film printing was reported in detail. To also experimentally confirm the behaviour and features of the actuator, the performance of three prototype devices were reported, two of which were built on steel substrate, while one was built on alumina. All units were 1st-order designs, and both the proper functioning and targeted displacement profile of the design were shown on the basis of data collected by laser vibrometer measurements.

Subject of future work remains the study of the behaviour of higher-order actuators built on the basis of the proposed staircase actuator, as well as a more detailed investigation of the advantage pertaining to the stress in the joint connecting to the load. In addition, the impact of the choice of material on both the dynamic behaviour and the resilience to stress in the joint would be of interest.

## Figures and Tables

**Table 1:** Table of design parameters of the 4th generation devices.

Parameter	Type 1	Type 2	Type 3
	(Alumina)	(Steel)	(Steel)
Substrate thickness $d_{S}$ (μm)	150	100	100
Beam width *L* (μm)	1450	2450	1450
Beam length *S* (μm)	10,550	9750	10,550
Electrode thickness $d_{e1}=d_{e2}$ (μm)	5	5	5
PZT thickness $d_{p}$ (μm)	10	10	10
Feature gap $g_{P}$ (μm)	350	350	350
